# Loneliness and Social Isolation Interventions: What Works, What Doesn’t, and What’s Next?

**DOI:** 10.1093/geroni/igaf122.192

**Published:** 2025-12-31

**Authors:** Kexin Yu, Chuwen Zhong, Matthew Smith

## Abstract

Loneliness and social isolation in older adult populations pose significant public health concerns. Despite numerous interventions, their effectiveness remains mixed, necessitating a critical examination of what works, what doesn’t, and why. This symposium brings together experts to explore diverse approaches to reducing loneliness, ranging from targeted interventions to broad public health strategies. Kexin Yu will present findings from a pilot online conversation intervention designed to enhance social connectedness and cognitive health among older Chinese immigrants, highlighting the role of culturally tailored digital solutions. Kimberly Van Orden will share results from a clinical trial on a psychotherapy/coaching program aimed at reducing loneliness, which used Ecological Momentary Assessment (EMA) and passive sensing to capture sensitive changes in aspects of loneliness, offering insights into psychological approaches and personalized support. Christina Victor will critically examine the challenges of loneliness interventions, discussing why many fail and how future efforts can be more effective. Finally, Roger O’Sullivan will explore a public health approach to addressing loneliness and social isolation, emphasizing population-level strategies and policy implications. Together, these presentations will provide a comprehensive discussion of loneliness interventions, addressing both individual and systemic factors. The session will engage attendees in a dialogue about the future of research, practice, and policy in fostering meaningful social connections among older adults.

